# Insights on the Role of Sialic Acids in Acute Lymphoblastic Leukemia in Children

**DOI:** 10.3390/ijms26052233

**Published:** 2025-03-01

**Authors:** Kimberley Rinai Radu, Kwang-Hyun Baek

**Affiliations:** 1 Department of Life Science, Graduate School, CHA University, Seongnam-si 13488, Gyeonggi-do, Republic of Korea; kiimrinai@gmail.com; 2 Department of Bioconvergence, Graduate School, CHA University, Seongnam-si 13488, Gyeonggi-do, Republic of Korea

**Keywords:** hematological malignancy, hypersialylation, sialic acids, Siglecs

## Abstract

Sialic acids serve as crucial terminal sugars on glycoproteins or glycolipids present on cell surfaces. These sugars are involved in diverse physiological and pathological processes through their interactions with carbohydrate-binding proteins, facilitating cell–cell communication and influencing the outcomes of bacterial and viral infections. The role of hypersialylation in tumor growth and metastasis has been widely studied. Recent research has highlighted the significance of aberrant sialylation in enabling tumor cells to escape immune surveillance and sustain their malignant behavior. Acute lymphoblastic leukemia (ALL) is a heterogenous hematological malignancy that primarily affects children and is the second leading cause of mortality among individuals aged 1 to 14. ALL is characterized by the uncontrolled proliferation of immature lymphoid cells in the bone marrow, peripheral blood, and various organs. Sialic acid-binding immunoglobulin-like lectins (Siglecs) are cell surface proteins that can bind to sialic acids. Activation of Siglecs triggers downstream reactions, including induction of cell apoptosis. Siglec-7 and Siglec-9 have been reported to promote cancer progression by driving macrophage polarization, and their expressions on natural killer cells can inhibit tumor cell death. This comprehensive review aims to explore the sialylation mechanisms and their effects on ALL in children. Understanding the complex interplay between sialylation and ALL holds great potential for developing novel diagnostic tools and therapeutic interventions in managing this pediatric malignancy.

## 1. Introduction

Sialic acids have become important players in the field of cancerous tumors [1]. Among the various types of sialic acids present in humans, N-acetylneuraminic acid (Neu5Ac) is the most common form, with other derivatives synthesized in the biosynthetic pathway [2]. For metastasis and tumor growth, cancer cells’ capacity to connect and communicate with cells inside the tumor microenvironment (TME) is essential. Therefore, improving the effectiveness of treatment requires an understanding of the basic activities of TME cells and cancer cells. The importance of integrating molecular and cellular biology into the standard medical curriculum, particularly the concept that ‘DNA makes RNA makes protein’ [3], underscores the previously underappreciated role of macromolecules, especially sugar chains or glycans [4]. Despite the complexity and difficulty of researching glycans, current advances have given prominence to the specialized subject of glycobiology, which acknowledges the various physiological and pathological interactions involving glycans, which are present in all biological systems [5].

This review will focus on a less commonly studied derivative of sugars known as sialic acids. These sugars are commonly observed at the ends of glycan chains on most cell types [6]. In vertebrates and “higher” invertebrates, they complement a great deal of cell surfaces and generate proteins that are crucial for monitoring and mediating a variety of pathogenic processes. Sialic acids have an assortment of structural and modulatory functions because of their hydrophilicity and negative charge, including acting as binding sites for a range of toxins and pathogens [7]. Scientists have recognized their pathogen-binding abilities through specific interactions involving sialic acids presented on defined underlying sugar chains in specific linkages. The hosts expressing it can experience detrimental effects [2].

Previous evidence indicates that sialic acid-binding proteins, discovered over the last few decades, possess a mechanism of ‘molecular mimicry’. This capability allows them to assist in evading host immunity by constantly changing to prevent pathogens from rapidly evolving through binding or mimicking them [6]. Additionally, these proteins have distinct functions in both health and illnesses, including acute lymphoblastic leukemia (ALL). The growth of aberrant, immature lymphocytes and their progenitors, culminating in the infiltration and replacement of bone marrow and other lymphoid organs, is the distinctive feature of ALL [8]. In children diagnosed with ALL, common symptoms include anemia, thrombocytopenia, neutropenia, as well as manifestations such as spontaneous bleeding, frequent infections, and persistent fatigue [9].

In general, the human immune system utilizes a sophisticated mechanism to destroy cancer cells. In certain cases, though, they become immune surveillance evaders through acquiring resistance to the antitumor response. Cancer immunotherapy has completely changed the oncology sector by providing a variety of techniques which employ the patient’s own immune system to fight cancer cells. These techniques include adoptive transfer immune cell manipulation and immune checkpoint-targeted therapy. Both approaches recognize and attack the malignancy attributed to DNA damage, which triggers uncontrolled growth of lymphoid cells, evasion of sialic acid detection, and subsequent systemic migration [10]. The majority of ALL cases occur in patients under the age of 18, making it a prevalent childhood malignancy that peaks between the ages of two and ten [11].

This review will also cover how cellular sialome is a physiologically active and dynamically changing component [12]. Sialylation is emerging as a significant factor against tumor progression, which is altering cellular sialylation patterns and currently serving as a modulator of chemotherapy effectiveness. By focusing on its intricate interplay with cancer treatment in the extracellular matrix (ECM) and immunosuppressive processes, recent years have seen Siglecs emerge as a family of immunoregulatory receptors known as glyco-immune checkpoints. The integration of immunoregulatory pathways, such as T cell immune checkpoints, into anti-cancer immunotherapies has shown promise [13]. In humans, a total of 15 Siglec members have been identified, including Siglec-2 (CD22), Siglec-4 (myelin associated glycoprotein, MAG), and Siglec-15 (CD33L3). These N-acetylated Siglecs are expressed on monocytes, neutrophils, natural killer (NK) cells, and B cells [14].

## 2. Sialic Acid

### 2.1. Chemical Structure

**Figure 1:**
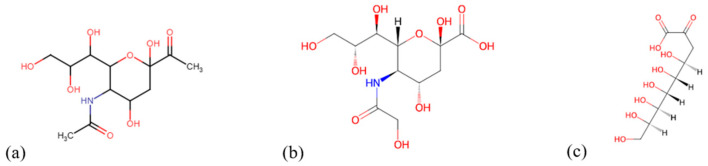
The basic structures of sialic acids. (**a**) N-acetylneuraminic acid (Neu5Ac), (**b**) N-glycolylneuraminic acid (Neu5Gc), and (**c**) 3-deoxy-d-glycero-d-galacto-2-nonulosonic acid (KDN). (Red indicates -O bonds, while blue indicates -NH bonds).

Sialic acids, also known as N-acetylneuraminic acids (Neu5Ac) (Figure 1), are 9-carbon carboxylated monosaccharides characterized in mammals. They are predominantly found at the outermost ends of N-linked and O-linked carbohydrate chains and in lipid-associated glycoconjugates, but are absent in plants [2]. The negatively charged and hydrophilic properties of sialic acid play pivotal roles in various normal and pathological processes. Functioning as binding sites, they interact with numerous pathogens and toxins, wherein pathogen-binding proteins recognize distinct sialic acid linkages—an evolutionary adaptation believed to have emerged among vertebrates to facilitate “molecular mimicry”, a process by which microbial pathogens adopt host sialic acids to evade the host’s immune system.

**Figure 2:**
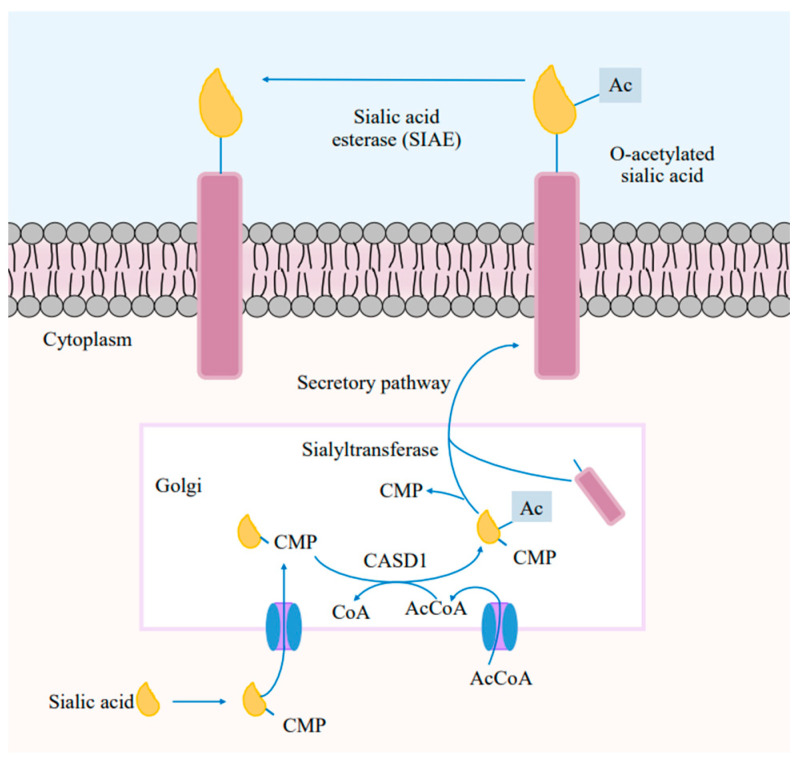
Schematic representation of the sialic acid biosynthesis pathway in mammalian cells.

Cell membrane glycomolecules form a dense network resulting in high diversity and dynamic variability [15]. Glycans are covalently conjugated to a protein core, attaining complex structures that resemble a forest within this network. The stereospecific production of glycosidic linkages and the monosaccharide units that control the spatial organization of glycans are what give these structures their complexity and roles. This changing terrain influences sialylation and produces distinct glycosylation patterns. Structural studies of these defined sequences (Figure 2) feature a wide expression of neuraminic acid derivatives, known as sialic acids. The attachment of sialic acids to the non-reducing ends of sugar chains represents the final step in glycosylation, resulting in enhanced microheterogeneity that influences the physical and biochemical properties of glycoconjugates [16].

### 2.2. Functional Features

Sialic acids can undergo further modifications, including O-acetylation, O-methylation, and O-lactylation at four hydroxyl groups [17]. Additionally, 3-deoxy-d-glycero-d-galacto-2-nonulosonic acid (KDN) is commonly found in vertebrates, although its presence is limited to lower vertebrates. The negative charge and hydrophilicity of sialic acids provide charge repulsion, avoiding unwanted interactions between different cells in blood circulation. Extended polysialic acid chains can also affect neuronal plasticity, the luminal surface of the vascular endothelium, and modulate the half-life of proteins during infectious phases with sialidase-expressing bacteria [10].

Their ubiquity and varied forms make sialic acids versatile molecules that participate in many physiological processes, particularly in biological recognition, cellular communication, adhesion, and migration. These processes are mediated by highly specific sialoglycans, which influence dimerization, activation, and autophosphorylation, resulting in changes that control cell proliferation and survival [18]. Proper sialylation patterns and the activities of sialyltransferases and sialidases are crucial for homeostasis, affecting cellular roles in nephrology, neurobiology, hepatology, cardiovascular physiology, reproductive processes, and immune response [19].

Pathogens exploit molecular mimicry by decorating themselves with sialic acids to evade the host immune system [20,21,22]. Among living things, the human brain has the largest concentration of sialic acid, which raises the possibility that various isomers of sialic acid levels are related to one another [23]. In Neu5Gc or N-glycolylneuraminic acid, the terminal sialic acid residue is linked to the glycolic acid unit through the hydroxyl group, a process catalyzed by CMP-N-acetylneuraminic acid (CMP-Neu5Ac) hydroxylase (CMAH) in animals. However, humans are missing the *CMAH* gene, resulting in the absence of Neu5Gc [24,25].

Among the Siglecs studied on NK cells, Siglec-7 and Siglec-9 have received particular attention, although other forms of Siglecs on NK cells have also been identified. Siglec-3 (CD33), expressed at low levels as an inhibitory receptor during early NK cell development, shows differential expression across various developmental stages in distinct tissues [26]. Interestingly, through facilitating macrophage polarization, its expression on tumor-associated macrophages (TAMs) accelerates the progression of cancer. Research suggests that the functional diversity of macrophages is strongly linked to their plasticity, as their functional phenotypes are influenced by various molecules within the tumor microenvironment [27]. As a result, interactions between NK cell-expressed sialoglycans and tumor cells can prevent tumor cell death.

In other sialylation machineries, various types of cancers are related to and described as markers of sialome-related tumorigenesis [12]. Reports show that in aberrant sialylation processes, among the four sialidases (Neu1, Neu2, Neu3, and Neu4), an enhanced expression of Neu3 is seen in malignant tissues, while the other enzymes tend to be reduced, leading to an accumulation of sialoconjugates [28]. Additionally, the family of glycosyltransferases plays a part in glycosylation patters in an ever-changing complex mechanism closely related to the pathology of cancer, such as ALL in children [29]. Therefore, the dysregulation of sialyltransferases involves genetic, epigenetic, transcriptional, and post-transcriptional regulations associated with glycosylation patterns [30].

## 3. General Functions of Sialic Acid

### 3.1. Roles of Sialic Acid in the Body

The structural composition of sialic acids encompasses multifaceted functions, including their role as glycoconjugates and ligands for lectins, enzymes, and antibodies [31]. Sialic acids are crucial in cell–cell recognition, communication, aggregation, protein interactions, and the development of bacterial and viral infections, tumor growth, and metastasis. They are significant contributors to immunology, cell signaling, reproduction, and the nervous systems [31]. Moreover, sialic acids stabilize red blood cells by preventing aggregation through their negative charge and hydrophilicity. They also play important roles in hormone regulation and reproductive function [32], as well as in the intricate development of both the central and peripheral nervous systems [33]. O-acetylation is prevalent across various species and can take place at positions 4, 7, 8, and 9 of the sialic acid structure. Other modifications, such as O-methylation, O-sulfation, and O-phosphorylation, also contribute to the extensive structural and physicochemical variability of sialic acids [34].

Increasing emphasis is being placed on the critical role of sialic acids in human pathophysiology. Specifically, altered sialylation patterns in various cancers and histological forms, particularly hypersialylated glycans, contribute to malignant cell behavior, tumor progression, and the ability of cancer cells to interact with surrounding normal tissue and infiltrating immune cells [11]. These changes enhance the resistance of cancer cells to the body’s immune response, establishing sialic acid-mediated immune evasion as a hallmark of numerous malignancies [16].

Furthermore, the biosynthesis of sialic acid begins with *N*-acetylmannosamine (ManNAc), which is derived from UDP-*N*-acetylglucosamine (UDP-GlcNAc) [35]. This process involves phosphorylation to produce ManNAc 6-phosphate (ManNAc-6P), followed by condensation of either ManNAc or MacNAc-6p with phosphoenolpyruvate to produce NeuNAc or NeuNAc-9P. The final form, CMP-sialic acid, is the activated form of sialic acid generated through the incorporation of cytosine triphosphate, CTP [35].

Recent studies have emphasized the role that sialylation plays in the development of cancer. Siglecs, which are mainly present on the surface of white blood cells, have immunological checkpoint inhibitory properties and are essential for immune-mediated detection of both self and non-self antigens [36]. Siglecs are part of the I-type lectin family within the immunoglobulin superfamily, with the ability to identify and attach to sialic acids present on glycoproteins and glycolipids [36].

#### 3.1.1. Sialic Acids Synthesis in Cancer

The biosynthesis of sialic acids usually undergoes alterations at different stages in cancer. The pathways involved consist of three main stages: the synthesis of Sia monomers, the transfer of the Sia, and the cleavage and recycling of Sia molecules. Various malignancy phenotypes are linked to the dysregulation of these processes, such as the activity of sialyltransferases (Sts) and cytidine monophosphate N-acetylneuraminic acid synthetase (CMAS), which modify cell surface sialylation (Figure 2) [37]. Inflammation is strongly implicated at all stages of cancer [38], and tumor progression and survival are exploited through inflammation, which mediates cancer cell invasion into secondary tissues. These cells then evade immune checkpoints, highlighting the intention of scientific efforts to disrupt the cycle of immune suppression.

A strategy depicting metabolic incorporation of unnatural components into cell surface glycoconjugates through pathways involves oligosaccharide biosynthesis. In this process, cells are given unnatural monosaccharide precursors, which are then converted by the biosynthetic machinery into new products that appear on the cell surface. It requires the biosynthetic enzymes to effectively process modified substrates in comparison to natural substrates. The sialic acid biosynthesis pathway has shown the capacity to accommodate unnatural substrates, making it a promising option for the metabolic engineering of cell surfaces.

#### 3.1.2. Different Roles of Sialic Acids

Sialic acid is a distinctive sugar component found at the outermost end of glycans on the surfaces of mammalian cells, as well as on extracellular glycoproteins and glycolipids. It has long been suggested that sialic acid could obscure underlying structures, but the precise mechanisms through which these structural alterations directly impact biological processes are still being discovered and experimented on [39]. The general understanding is that twenty different sialyltransferases facilitate the addition of sialic acid to underlying glycans in α2,3, α2,6, or α2,8 linkages, while four mammalian sialidases catalyze its removal [40].

Recent reports, such as those focusing on the sialyltransferase ST6GAL1—responsible for adding Sia(α2,6) to the Gal(β1,4)GlcNAc-R termini of glycans—have revealed surprising diversity in sialylation mechanisms within biological systems. ST6GAL1 is implicated in various clinical conditions such as stress, inflammation, atherosclerosis, alcoholism, and several types of cancer, including colon, breast, and pediatric acute leukemia [41]. ST6GAL1 is also involved in inhibiting the differentiation of human pluripotent stem cells. Typically, sialyltransferases are located within the intracellular ER-Golgi secretory network, where the modification of glycoconjugates occurs during biosynthetic transit. The full-length of ST6GAL1 exhibits the characteristic features of a typical glycosyltransferase. The concept of ’Extrinsic Sialylation’ suggests that extracellular sialyltransferases maintain their enzymatic activity and directly add sialic acid to secreted or cell-surface glycoproteins via a pathway that bypasses the Golgi apparatus [42].

The proposal identifies different extrinsic sialylation known as the endocrine, paracrine, and autocrine, highlighting the significance of the physical relationship between secreting and target cells. According to this understanding, alterations in secreted sialyltransferase levels can swiftly affect sialic acid-dependent functions on a local and systemic level. This is consistent with research showing that blood sialyltransferase levels change in response to illness or stress, suggesting that extrinsic sialylation may have biologically significant effects. In contrast, “Intrinsic Sialylation” refers to the traditional mechanism where cell-autonomous sialyltransferases work within the intracellular ER-Golgi network [43].

### 3.2. Hypersialylation and Cancer

**Figure 3:**
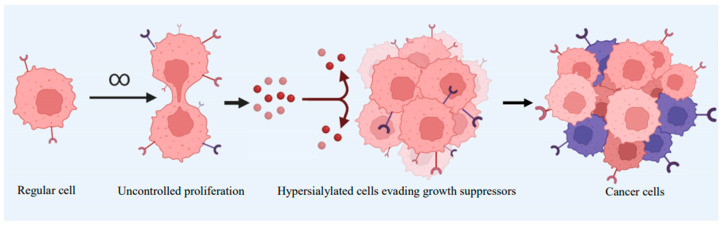
Depiction of hypersialylation from a regular cell growing beyond its regular cell cycle into cancerous cells of several heterogeneity that enables them to increase chances of survival. Created with BioRender.com.

Hypersialylation is commonly observed in tumor tissues compared to their corresponding normal counterparts [44]. Elevated levels of total sialic acid in serum or glycolipid-bound sialic acids (Figure 3) have been observed in various cancers, including ovarian, colorectal, and breast cancer, with a particular emphasis in this review on leukemia [45]. Polysialic acid, a specific type of sialic acid, is highly expressed in several cancer types, including glioma, neuroblastoma, and lung cancer. The increased levels of sialic acids in tumor cells primarily result from distinct metabolic fluxes and the aberrant expression of sialyltransferases/sialidases [46].

In the context of critical cancer progression, sialic acids impact cellular recognition, signaling, molecular trafficking, migration, and adhesion. Aberrant glycosylation on the cancerous membrane alters glycoprotein expression, promoting tumorigenesis [47]. As cell migration and the metastatic potential of malignant cells are crucial, the overexpression of sialic-acid-terminated glycans results in alterations of the immune system and antibody functions, facilitating tumor aggressiveness [11,48]. The biological tuning of proteins through glycosylation machinery allows for selective functions, such as mediating cell–matrix interaction, cell adhesion, host–pathogen recognition, and protein folding [11].

Sialic acid residues positioned at the tips of glycans play a critical role in cellular processes involving cell-to-cell contact. Increasing evidence demonstrates significantly elevated levels of sialic acids on cancer cells compared to non-transformed cells [49]. Research into the mechanisms is ongoing, with preclinical models aiming to decrease sialic acid on cancer cells or block key interactions between them and relevant receptors on myeloid cells, which correlate with inflammatory environments favorable to cancer cells [50,51]. Given that some situations may benefit from hypersialylation without explicitly involving inflammation, it is crucial to thoroughly investigate the relationship between inflammation and hypersialylation.

## 4. Acute Lymphoblastic Leukemia (ALL)

### 4.1. Brief Background on ALL

ALL is a heterogeneous hematological malignancy that predominantly affects pediatric populations. It is characterized by the uncontrolled proliferation of immature lymphoid cells in the bone marrow, peripheral blood, and other organs. Extensive research and modern approaches have led to the recognition of multiple ALL subtypes based on the presence of specific somatic genetic lesions, which subsequently influence therapy selection and overall prognosis.

Studies on ALL have demonstrated its close associations with immunity and infections. Children with ALL are particularly susceptible when their immune systems are suppressed during childhood, leading to immune-modulated responses [52]. There are indications that ALL is a relatively common form of cancer among children under 5 years of age, with a decline in risk during the mid-20s and a gradual increase after the age of 50. Notably, 40% of cases occur in adults, leaving children within a vulnerable age range [53]. The incidence of childhood ALL is increasing worldwide, suggesting that its prevalence is influenced not only by hereditary factors but also by environmental factors such as exposure to ionizing radiations, chemotherapeutic drugs, genetic syndromes, and immunological factors [54].

On top of that, the rate of survival remains substantially lower in low- and middle-income countries (LMICs) due to high rates of treatment-related toxicity, treatment discontinuation, and other challenges [55]. To ensure a global standard is in place, the modified version of the standard Berlin–Frankfurt–Munich (BFM) protocol is suitable for pediatric ALL to achieve a balance between toxicity and positive response rate [56]. Unfortunately, although recovery is promising, ALL is still considered the second leading cause of mortality among children aged 1 to 14 [57].

### 4.2. Molecular Mechanisms Leading to ALL

Some cases of ALL in children are associated with the translocation t(9;22), which leads to the Philadelphia chromosome abnormality. Philadelphia chromosome-positive ALL (Ph+ ALL) is known for its poor prognosis, with long-term survival rates of only 10–20% [58]. The *BCR::ALB1* fusion oncoprotein, resulting from the reciprocal translocation t(9;22), possesses intrinsic tyrosine kinase activity. This activity upregulates various cell cycle signaling pathways, including RAS/RAF/MEK/ERK, PI3K/AKT/mTOR, and JAK/STAT. These pathways correlate with the activation of other tyrosine kinases, such as SRC family members (e.g., LYN, HCK) and MYC, leading to dysregulated cell proliferation and reduced apoptosis in lymphohematopoietic cells. Targeting this protein through molecular therapeutic methods dysregulates tyrosine kinase activity. This is due to the underlying mechanism of the respective transcription factor genes in cell proliferation and tumorigenesis. The primary genomic risk factors encompasses chromosomal translocations, gene fusions, and rearrangements including *BCR:ABL1* t(9;22), *TVF3::HLF* fusion t(17;19), KMT2A rearrangement t(4;11), MEF2D rearrangement t(1;19), TCF3:HLF fusion, mixed-phenotype acute leukemia (MPAL), early T-cell precursor acute lymphoblastic leukemia (ETP-ALL), and intrachromosomal amplification of chromosome 21 (iAMP21) [59].

The B-cell acute lymphoblastic lymphoma (B-ALL) in children is characterized by the rapid proliferation of poorly differentiated lymphoid progenitor cells. In the bone marrow where these cells are assembled, tumorigenesis involves several abnormal gene expressions such as *BCR-ABL-1*, PI3K and *TEL-AML1* [60]. These lymphoid cells developed from pluripotent hematopoietic stem cells in the bone marrow and primarily differentiated from the common pro-B cells, pre-B cells, and mature B cells. The cell transduction process is usually managed throughout the maturation process [61].

Apart from mutations in transcription factors experienced during hematopoietic development to B-ALL, genetic susceptibility through germline predisposition is also acknowledged in this review. The observation comes from the first report of *RUNX1* following an autosomal-dominant inherited from a single altered copied gene [62]. Regulated genes in these conditions affect DNA damage recognition, apoptosis, cell differentiation, signal transduction and ribosome function. When these cellular processes are interrupted, uncontrolled metastasis occurs. These germline alleles affect a wider range of hematopoietic malignancies to full-blown leukemia. Progression often follows a distinct pattern depending on the germline predisposition. It is common to observe acquired somatic mutations within leukemic cells such as germline SAMD9/SAMD9L, leading to a loss of deleterious germline allele. However, these findings suggest a conundrum in understanding the relationship between genetic factors and extrinsic exposures promoting malignant transformations [62].

### 4.3. Clinical Trials and Treatments

Treatment of ALL is an intricate and prolonged process, requiring multi-agent chemotherapy, with asparaginase playing a crucial role in the regime [63]. Recognizing that leukemic cells require external sources of asparaginase for growth, inhibiting this enzyme has the potential to eradicate leukemic cells [64]. Recent studies have suggested the use of targeted agents, such as tyrosine kinase inhibitors, for children expressing the BCR-ABL oncoprotein or exhibiting mutations in the JAK-STAT pathway [65]. Furthermore, research has identified genes associated with predisposing conditions targeted by somatic mutations in ALL blasts, including PAX5, IKZF1, ETV6, and PTPN11, as mentioned in the previous section regarding gene regulation [66].

Cancer immunotherapy, mainly checkpoint-targeted therapy and the adoptive transfer of engineered immune cells, has revolutionized oncology by leveraging the patient’s immune system [67]. Checkpoint inhibitors, like programmed cell death ligand 1 (PD-L1) and cytotoxic T-lymphocyte-associated protein 4 (CTLA-4) antibodies, along with agonists of costimulatory molecules, bypass inhibitory pathways to activate immune responses [68]. Despite successes in clinical trials, challenges include low response rate, high costs, and nonspecific toxicity [68,69,70]. Other methods include adoptive cell transfer, genetically engineered chimeric antigen receptor (CAR)-T cells, and multipotent mesenchymal stem cells, which manipulate cytokines and other characteristics of anti-cancer cells [71].

While previous immunotherapies focused on stimulating adaptive immunity by revitalizing T cell responses, more recent studies highlight the critical role of innate immune checkpoints expressed on antigen-presenting cells (APCs) in immune evasion. Targeting macrophages, monocytes, dendritic cells (DCs), and NK cells—the first line of the body’s immune defense—is crucial. ALL cells evade clearance by macrophages via overexpressing anti-phagocytic membrane proteins [66] like cluster of differentiation 47 (CD47), cluster of differentiation 24 (CD24), PD-L1, β-2 microglobulin (β2M) subunit of the marker histocompatibility class I complex (MHC-I), GD2, and stanniocalcin 1 (STC1).

Phagocytosis, mediated by phagocytic receptors, triggers extensive cytoskeleton remodeling facilitated by “eat me” signals [72] such as SIRPα, Siglec-10, and LILRB1, is highly expressed. Additionally, the expression and function of CD22 and its synthase, cytidine monophosphate N-acetylneuraminic acid synthetase (CMAS), involve binding proteins to its cytoplasmic tail in response to B-cell antigen receptor signaling. This process, involving PTPN6 encoding SHP-1 and spleen tyrosine kinase (Syk), and others, play a significant role in ALL pathogenesis [73]. Further information will be discussed below on the roles of Siglecs against ALL cells.

## 5. Siglecs Against ALL

### 5.1. Types of Siglecs

**Figure 4:**
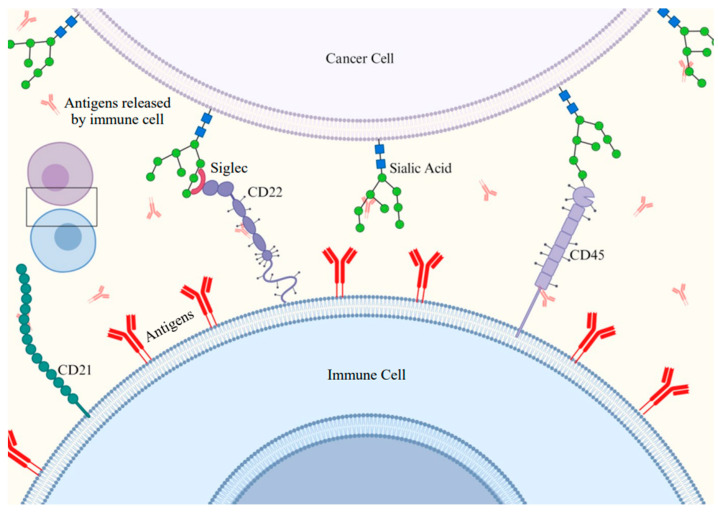
Interaction between immune cells (T- and B-cells) and cancer cells within the immunosuppressive tumor microenvironment (TME), highlighting the attachment of Siglecs to signals from cancel cells and MHC antigens as recognition sites. Created with BioRender.com.

Siglecs, also known as sialic acid-binding immunoglobulin-like lectins, are cell surface proteins that possess the ability to bind to sialic acids [74]. The discovery of sequence similarities among sialic acid-binding proteins, including sialoadhesin, CD22, CD33, and myelin-associated glycoprotein (MAG), led to the identification of a subgroup later coined as Siglec-1 through Siglec-4 [75]. The greater part of Siglecs is engaged in cell–cell adhesion, whereas a small number also serve as activating receptors (Figure 4) [76]. B-cell precursor acute lymphoblastic leukemia (BCP-ALL) is the most common type of cancer in children and is also frequently diagnosed in adults [77].

The sialic acid–Siglec axis is fundamental in mediating cell adhesion and signaling, as well as for moderating self-recognition and non-recognition. An immunoglobulin-like inhibitory motif (ITIM)-containing inhibitory Siglecs controls the immunological and non-immune responses in the TME and affects multiple aspects of cancer progression. Reports have shown that the TME encourages abnormal secretion of Sia, leading to an upregulation of Siglec expression to infiltrate immune cells. Thus, tumors with dysregulated Sia-Siglec axis further contribute to immunosuppressive cell signal transduction, concerns tumor growth and avoidance of immune escape. In hypersialylated cancer cells, the binding of Siglec-7 on NK cells leads to reduced immune responses and irregular cell behavior [78].

As for a general understanding, there are 15 members of the Siglec family in humans, variously expressed on white blood cells, with an increasing number of examples showing Siglec expression outside the immune cell lineage [79]. Their presence and inhibitory functions are primarily due to ITIMs on their cytoplasmic tails. These motifs can be phosphorylated under the appropriate physiological or pathophysiological circumstances, thereby dampening immune cell signaling. However, Siglec14–16 do not contain ITIMs, but instead a lysine residue in their transmembrane region, which facilitates pairing with immunoreceptor tyrosine-based activation motif (ITAM) containing adaptor proteins [76]. The ability of Siglecs to recognize sialic acid, which is often upregulated in cancer, and to alter immune cell responses is an active area of research with numerous proposed mechanisms [80].

### 5.2. Siglec Checkpoint and Interplay Resulting in Immunotolerance

CD22 expression is detected during the early stages of B cell development in the bone marrow and spleen [81]. Furthermore, CD22 is expressed on B lymphocytes isolated from various lymphoid compartments. Activated B lymphocytes and precursor B-cells exhibit upregulated CD22 expression on their cell surface and in their cytoplasm [82]. CD22 acts as a key component of the B cell receptor (BCR) complex, interacting with proteins of the Siglec family to provide specificity and regulate BCR signaling. CD22 is categorized as an adhesion molecule within the immunoglobulin superfamily, with its extracellular region comprising seven Ig-like domains. A detailed model indicates that CD22 works as a co-receptor with Siglec-G to negatively fine-tune BCR signaling [83]. These signaling molecules involved include BCR cross-linking, phosphorylation, activation of tyrosine phosphatases and more.

CD33 is expressed by NK cells derived from blood when stimulated in vivo; however, the extent of its roles on NK cells remains relatively unexplored. Previous studies suggest its possible role in inhibiting signals from activating receptors. Nonetheless, different tissue contexts and the multifaceted roles performed by Siglec cells should also be considered. The high specificity of CD33 distribution and the differences in the structure of intracellular signaling domains relate to interactions between receptors and ligands, determining whether signaling pathways are activated or suppressed [84].

Siglec-7 and Siglec-9 are predominantly studied because most experiments are performed with NK cells derived from peripheral blood [74]. These NK cells and neutrophils are innate immune cells capable of immediate responses toward killing virus-infected or transformed cells (Figure 2). Nicoll et al. (2003) proposed that the inhibitory NK-mediating killing of cancer cells is possible when cancer cells upregulate the expression of sialic acid-containing Siglec-7 ligands and engage them onto NK cells. Evidence shows that the hypersialylation of cancer cells inhibits the response of NK cells predominantly via Siglec-7 [85].

Siglec-9 or its murine equivalent Siglec-E on neutrophils dampens the response of neutrophils to cancer cells. This exploitation by cancer cells overexpressing sialic acid on their surface is similar to the effect of Siglec-7 on NK cells. Evidence has shown that polymorphisms in Siglec-9 are able to partially abrogate sialic acid binding, providing better survival in patients with cancer [86]. An established murine tumor model proposed that Siglec-9 with its sialic acid-containing ligands on cancerous cells inhibited the skewing of macrophages, validated on tumors implanted into mice [87].

#### Medicinal Therapeutic Reports Against Host Pathogens

To date, Rituximab, a chimeric anti-CD20 monoclonal antibody, is commonly used for anti-myelin-associated glycoprotein (anti-MAG) therapy. Studies have reported that Rituximab, when combined with chlorambucil or bendamustine, induces higher response rates and longer progression-free survival [88]. It has been observed that anti-MAG antibodies are pathogenic, and repeated cycles of Rituximab treatment have shown effectiveness, although progressive loss of efficacy has been noted [89,90]. This is because the presence of anti-MAG antibodies is associated with neuropathy-associated IgM monoclonal gammopathy, suggesting that MAG molecules serve as accessible antigens located on the membrane surface [91]. Specifically, myelin-associated glycoprotein (Siglec-4) exhibits a significantly higher binding affinity, 500–10,000-fold greater, toward mono- and di-sialated derivatives of the O-linked T-antigen (Galβ(1–3)-GalNAcαOThr) compared to α-methyl-NeuAc [92].

## 6. Drug Resistance in Cancer

An emerging area of research focuses on targeting immune cells responsible for inflammation, especially those associated with standard immune checkpoint inhibitors [93]. Overcoming drug resistance remains a significant challenge, as the molecular profiles of cancers are not fully understood, preventing a complete cure. Concurrent findings indicate that cancer cells develop resistance to chemotherapy through various mechanisms, adapting at the cellular level and resulting in a poor response to therapy.

Genetic factors and the acquired use of chemotherapeutic agents contribute to this resistance, with multi-drug-resistant (MDR) cancer therapy posing one of the most formidable obstacles for patients and researchers alike. Despite advancements in medical research and development, tumors with this characteristic can acquire distinct structures and modes of action, rendering them resistant to a broad spectrum of treatments [12]. MDR can also arise from extrinsic mechanisms such as insufficient blood flow through the tumor structures, poor vascularity, or inability of drugs to reach the cells due to decreased pH, resulting in hypoxia and lactic acid accumulation [94].

In the context of tumor cell sialylation, previous reports suggest that several sialyltransferases are involved in the PI3K/AKT pathway. MDR phenotypes have shown a positive relationship between sialyltransferase levels and this pathway. Targeting and inhibiting this pathway can mitigate adverse effects caused by the overexpression of sialyltransferases such as ST8SIA4 or ST6Gal-I, both of which play significant roles in the Pi3K/Akt pathway. Knocking down these sialyltransferases could hinder the growth, proliferation, and migration of aggressive prostate cancer cells while reducing levels of several pathway components [95].

MDR represents an aggressive threat, with constant modifications to invasiveness, metastasis, and proliferation in downstream pathways potentially setting back research and medical approaches. These resistances are influenced not only by overexpression but also by sialidases. An experiment by Nath et al. (2018) demonstrated that increased membrane-bound Neu2 expression in drug-resistant pancreatic ductal adenocarcinoma (PDAC) cells was linked to intensified apoptosis via Fas activation, a weakened PI3K pathway, and decreased invasiveness, metastasis, and cell proliferation [96].

Clinical observations by Yin (2017) suggest that an increased infiltration of tumor-associated macrophages (TAMs) is associated with chemoresistance in patients with colorectal cancer, indicating that immune cells the effect of TAMs on cellular drug sensitivity [97]. Additionally, researchers should consider the impact of epigenetic modulation on the response of malignant cells to therapy. Evidence indicates that downregulated miR-331-5p and miR-27a are related to resistance to the chemotherapy drug doxorubicin, leading to relapse in leukemia [98].

## 7. Current Clinical and Approved Treatments

**Figure 5:**
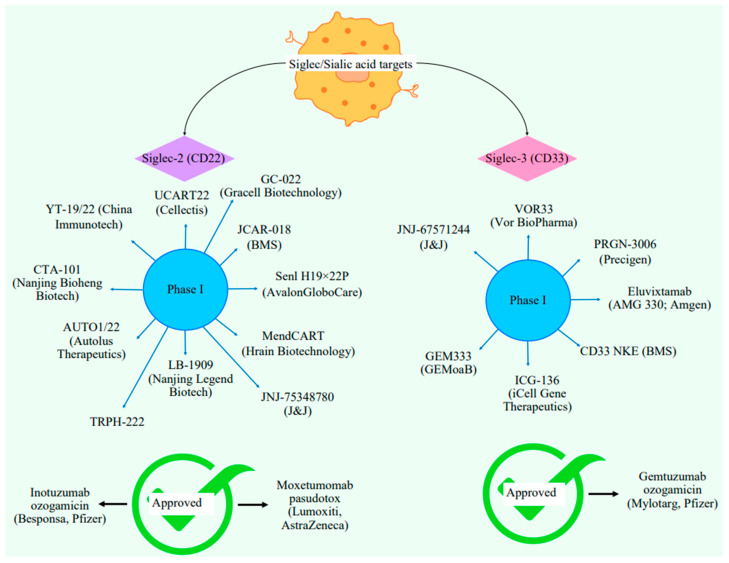
Recent developments on targeting Siglecs as tumor-associated markers.

Antibody-based therapeutics are increasingly focused on targeting malignant immune cells, utilizing antibody-tethered cytotoxic functions in the form of antibody-drug conjugates (ADCs), anti-Siglec bispecific T-cell engagers (BiTE), and the better-known chimeric antigen receptor (CAR) T-cell therapies [98]. A summary of the agents targeting Siglecs are provided in Figure 5.

ADCs are particularly effective for delivering toxins within a specific subset of immune cells, as Siglecs undergo endocytosis upon ligand binding [99]. Siglec-3, highly expressed and enriched on AML cells, serves as a lineage marker for myeloid cells. Additionally, Siglec-2 expressed on B cells is targeted by anti-Siglec-2 ADCs in the treatment of B-cell leukemia and lymphoma [100]. ADC therapies work by targeting myeloid cells, taking advantage of hypersialylation, and causing damage to DNA. These approaches have been used for leukemia, other than hairy cell leukemia, demonstrating the potential they hold as important targets for cancer therapy [101].

Advancements have also led to the development of Siglec-targeting BiTEs and CAR T-cell technologies [102]. BiTEs efficiently eliminate cancer cells by engaging cytotoxic T cells through two single-chain variable fragments linked by a flexible linker. One fragment targets the T cell surface marker CD3, while the other targets a specific antigen on the cancer cell, leading to T cell-mediated apoptosis [103].

CAR T-cell provides potent and highly specific targeting, although effective management of systemic immune toxicity is crucial. Clinical studies in the early phases indicate insufficient data regarding whether CAR T-cell therapies enhance efficacy or improve the therapeutic index compared to ADCs [104]. The mechanisms behind CAR T-cell therapy involve Siglec-2 targeting and hematopoietic toxicity, though the exact effects are not fully understood [105]. A recent CAR T-cell therapy has been developed to target Siglec-6, expressed on AML cell lines. Preclinical and in vitro studies have demonstrated significant antileukemia activity, suggesting a potential alternative to allogeneic hematopoietic stem cell transplantation [106].

### Immune Therapies Safe for Children

By targeting the novelty of hypersialylation as an immunomodulatory therapeutic strategy, especially for use against ALL in children, the sialic acid mimetic Ac(5)3F(ax)NeuAc acts as an inhibitor and has shown anti-tumorigenic properties in mice. It reduces sialylation, thereby enhancing NK cell and CD8^+^ T cell infiltration and reducing immunosuppressive regulatory T-cells and myeloid cells [80]. As previously mentioned, the use of monoclonal antibodies like rituximab is one of the promising treatments approved for use with ALL. Additionally, the US FDA has approved bi-specific antibodies (blinatumomab), antibody-drug-conjugates (inotuzumab ozogamicin), and chimeric antigen receptor (CAR) T-cells (tisagenlecleucel and brexucabtagene autoleucel) [72].

Reports from Queudeville and Ebinger (2021) revealed that children with B-cell ALL achieve histological complete remission with negative measurable residual disease (MRD) [107]. However, adverse effects were primarily noted in adults, including immune effector cell-associated neurotoxicity syndrome (ICANS), which is rare, and relapses after six months [108]. Many clinical trials, as shown in Figure 5, have shown promising improvements in progression-free survival. From a pharmacodynamic perspective, targeting T-cells to CD19-positive B-cells induces the production of adhesion molecules, cytokines, perforins, and granzymes, leading to programmed cell death and production and the rapid multiplication of associated T-cells [85].

Though immunotherapies utilizing immune cells of the body are generally safe, some concerns need to be addressed. When providing these treatments, dosing based on age and body weight should be considered [109]. Adverse effects such as cytokine release syndrome, neurotoxicity, cytopenia, and infections should be monitored. Tumor lysis syndrome (TLS) is a potential risk, especially in patients with a large leukemia burden during the treatment phase. Children with B-cell ALL are considered high-risk for developing TLS, which can range from arrythmias caused by hyperkalemia and hypercalcemia to kidney failure from uric acid buildup, necessitating preventive measures [110]. Other rare adverse effects in children with ALL include constipation, hyperglycemia, hypokalemia, hypophosphatemia, and pancreatitis [111]. Despite these challenges, ongoing and future studies are essential to assess the optimal timing and implementation of immunotherapies in ALL.

## 8. Conclusions and Further Prospects

Hypersialylation, a common phenomenon in tumors, increases their potency and prolongs their lifespan, significantly contributing to tumor progression. The aberrant sialylation process plays a pivotal role in this progression. Targeting these sialylations provides a platform for the development of novel therapeutic strategies and opens the door to new therapeutic opportunities. This review summarizes promising treatments that rely on inhibitors, antibodies, and agents targeting Siglecs. Furthermore, novel approaches are emerging to improve the detection of these altered sialylation patterns, which can now be customized to suit individual patient needs.

## Data Availability

Not applicable.
